# Quack Advertisements

**Published:** 1910-12-17

**Authors:** J. Inman Langley

**Affiliations:** "Lamorna," 40 Swanswell Terrace, Coventry


### Editor's Letter-Box.

### QUACK ADVERTISEMENTS.

*To the Editor of* The Hospital.

Sir,—The enclosed advertisement is from the Christmas Number of a popular illustrated magazine. What a tempting vista this absurd advertisement must open up to the mental vision of the ignorant and credulous. Imagine yourself "released for ever from the worry of job hunting," and, moreover, "advanced financially and socially to an equality with the best professional class of people, etc.," all on such beautifully simple and easy terms.

It is incomprehensible to me how a reputable publication can permit such a "thing" to have house-room. Is it not obvious that the object in view is simply to swindle poor fools out of their hardly earned money?

I am, Sir, your obedient servant,

J. Inman Langley.

"Lamorna," 40 Swanswell Terrace, Coventry,

December 7.

[The advertisement our correspondent encloses is not likely to deceive any professional man. As to the ethical points it raises, there is at present no possibility of limiting or regulating the insertion of similar advertisements in proprietary papers. All the members of the profession can do is to educate the public in the matter by pointing out the absurdity of these claims.—Ed. The Hospital.]

